# Tap water use amongst pregnant women in a multi-ethnic cohort

**DOI:** 10.1186/1476-069X-8-S1-S7

**Published:** 2009-12-21

**Authors:** Rachel B Smith, Mireille B Toledano, John Wright, Pauline Raynor, Mark J Nieuwenhuijsen

**Affiliations:** 1 Department of Epidemiology and Public Health, Imperial College London, London, W2 1PG, UK; 2 Bradford Institute for Health Research, Bradford Royal Infirmary, Bradford, BD9 6RJ, UK; 3 Center for Research in Environmental Epidemiology (CREAL), IMIM, CIBERESP, 08003, Barcelona, Spain

## Abstract

**Background:**

Studies of disinfection by-products in drinking water and measures of adverse fetal growth have often been limited by exposure assessment lacking data on individual water use, and therefore failing to reflect individual variation in DBP exposure.

**Methods:**

Pregnant women recruited to the Born in Bradford cohort study completed a questionnaire which covers water exposure. Information was collected on water consumption, showering, bathing and swimming. Water exposure data from a subset of 39 women of the cohort are described here.

**Results:**

Mean total tap water intake was 1.8 l/day, and women on average spent 146 minutes per week showering and bathing. Most tap water intake occurred at home (100% for unemployed, 71.8% for employed). Differences between age groups were observed for total tap water intake overall (p = 0.02) and at home (p = 0.01), and for bottled water intake (p = 0.05). There were differences between ethnic groups for tap water intake at home (p = 0.02) and total tap water intake at work (p = 0.02). Total tap water intake at work differed by income category (p = 0.001). Duration per shower was inversely correlated with age (Spearman's correlation -0.39, p = 0.02), and differed according to employment status (p = 0.04), ethnicity (p = 0.02) and income (p = 0.02).

**Conclusion:**

This study provides estimates of water exposure in pregnant women in a multi-ethnic population in the north of England and suggests differences related to age, employment, income and ethnicity. The findings are valuable to inform exposure assessment in studies assessing the relationship between DBPs and adverse birth outcomes.

## Background

Disinfection by-products (DBPs) are formed, when the added chlorine reacts with natural organic matter and/or bromide ions in the water [1]. Humans can be exposed to DBPs in drinking water by ingestion, or by inhalation and dermal absorption during activities such as showering [2]. There is some evidence to suggest that exposure to DBPs during pregnancy may be related to measures of compromised fetal growth, e.g. term low birth weight, or intra-uterine growth retardation [3,4], however findings are inconsistent and the evidence remains inconclusive. A major limitation in previous studies has been crude or incomplete exposure assessment; in particular, studies have often ignored individual variation in water use, therefore ignoring a potential source of variation in DBP exposure.

We are investigating the relationship between DBPs and measures of fetal growth in the Born in Bradford birth cohort [5]. We aim to improve on previous exposure assessment, by generating personalised DBP exposure estimates for each woman in the cohort during her pregnancy. At the area level, we have routinely collected information on trihalomethane concentrations in tap water supplied by the local water company, and as part of the HiWATE project [6] we have also conducted extra tap water sampling in the study area for non-trihalomethane DBPs. At the individual level, our exposure assessment involves evaluating exposure to water amongst pregnant women in the cohort. In this paper we describe patterns of water exposure within a subset of the cohort.

## Methods

Born in Bradford is a prospective multi-ethnic birth cohort in the north of England which is recruiting 10,000 mother and baby couplets between 2007-2010. Pregnant women are recruited to the cohort at approximately 28 weeks gestation. At recruitment detailed questionnaires are administered by bilingual researchers collecting data on the mothers' lifestyle, environment, ethnicity and health. Questions include water exposures: consumption of tap water, bottled water, tea, coffee, and squash at home, work/college, or elsewhere, water filtering habits at home and work, and showering, bathing and swimming habits. As part of a nested validation study 56 women were recruited from the main cohort during March and May 2008. The aim of the nested study was to collect detailed information which could be used to validation exposure estimates to DBPs and air pollution for the main cohort. To be eligible for the nested study women had to be able to speak and read English. Out of 166 eligible women, 56 (33.7%) agreed to take part. 12 women withdrew and 5 failed to complete the study, leaving 39 women. As part of this nested study we were provided with an extract of baseline questionnaire data for these 39 women by the Born in Bradford study, in advance of completion of the dataset for the main cohort for which recruitment is still ongoing. We analysed the baseline questionnaire data on this subset to provide descriptive statistics of water use, which are reported in this paper. Analysis was performed using R 2.4.1 [7]. Consumption was reported in cups or glasses per day (cup/glass assumed to be 200 ml), and converted into litres for analysis. Total tap water intake was calculated by summing tap water, tea, coffee and squash intakes. Total fluid intake was calculated by also including bottled water. When analysing by ethnicity, categories were collapsed to give 3 subgroups: White (incorporating White British and White Other), South Asian (incorporating Pakistani and Indian), and Other (incorporating Black or Black British and All Other), because numbers were small, and for employment subgroups, subjects on maternity/sick leave were kept with the employed group. The Born in Bradford study and the nested study were approved by the Bradford Research Ethics Committee.

## Results

### Demographics

Mean age of subjects was 29.7 years with just over half of the women employed (Table 1). A sizeable proportion of the women were educated to degree level (35.9%). 48.7% were of White British origin and 38.5% were of Pakistani origin. Income levels varied and only 10.3% reported currently smoking.

**Table 1:** Demographic characteristics

		Nested subset		Main cohort	
Characteristics		n	%	n	%
**All**		**39**	100.0	**4070**	100.0
					
**Age**	<20	**2**	5.1	**302**	7.4
	20-24	**4**	10.3	**1088**	26.7
	25-29	**13**	33.3	**1317**	32.4
	30-34	**15**	38.5	**839**	20.6
	35-39	**4**	10.3	**450**	11.1
	≥40	**1**	2.6	**73**	1.8
	Missing data			**1**	0.02
					
**Marital Status**	Married	**31**	79.5	**2864**	70.4
	Single	**8**	20.5	**1198**	29.4
	Missing data			**8**	0.2
					
**Highest Educational Qualification**	None	**4**	10.3	**698**	17.1
	O level/GCSE or A level	**13**	33.3	**1389**	34.1
	Degree	**14**	35.9	**827**	20.3
	Other (e.g. NVQ)	**8**	20.5	**1094**	26.9
	Don't know			**53**	1.3
	Missing data			**9**	0.2
					
**Employment status**	Employed	**20**	51.3	**1624**	39.9
	Unemployed	**18**	46.2	**2258**	55.5
	Maternity/Sick leave	**1**	2.6	**184**	4.5
	Missing data			**4**	0.1
					
**Parity**	0	**14**	35.9	**1587**	39.0
	1	**15**	38.5	**1198**	29.4
	2	**7**	17.9	**657**	16.1
	3+	**3**	7.7	**528**	13.0
	Missing data			**100**	2.5
					
**Household Income**	<£20,000	**15**	38.5	**1876**	46.1
	£20,000-40,000	**14**	35.9	**953**	23.4
	>£40,000	**7**	17.9	**332**	8.2
	Don't know	**3**	7.7	**846**	20.8
	Not stated/missing			**63**	1.4
					
**Ethnicity**	White British	**19**	48.7	**1573**	38.6
	White Other	**1**	2.6	**96**	2.4
	Pakistani	**15**	38.5	**1873**	46.0
	Indian	**1**	2.6	**159**	3.9
	Bangladeshi	**0**	0.0	**94**	2.3
	Any other Asian origin	**0**	0.0	**40**	1.0
	Black or Black British	**1**	2.6	**105**	2.6
	Mixed	**0**	0.0	**67**	1.6
	All Other	**2**	5.1	**59**	1.4
	Not stated/missing			**4**	0.1
					
**Smoking**	Current smoker	**4**	10.3	**562**	13.8
	Past smoker	**8**	20.5	**612**	15.0
	Never smoker	**27**	69.2	**2896**	71.2

### Water consumption

#### Overall

Mean total tap water intake across all locations was 1.8 l/day, whilst total fluid intake was 2.1 l/day (Table 2(a)). Tap water consumption (cold tap water and tap water based beverages) represented 84.3% of all fluid intake. For unemployed women, 100% of tap water intake occurred at home. For employed women 71.8% of tap water intake occurred at home, and 28.2% at work.

**Table 2:** Summary of water exposures

	2a: Water Consumption	Mean	Min	Percentile Distribution			Max	Consumed	
						
	Variable			0.25	0.50	0.75		n	%
**HOME**	Tap water (filtered and unfiltered) (l/day)	**0.7**	0.0	0.2	0.6	0.8	2.6	32	82.1
	
	Filtered tap water (l/day)	**0.2**	0.0	0.0	0.0	0.0	2.0	8	20.5
	
	Unfiltered tap water (l/day)	**0.5**	0.0	0.0	0.4	0.8	2.6	24	61.5
	
	Tea (l/day)	**0.3**	0.0	0.0	0.2	0.4	2.4	22	56.4
	
	Coffee (l/day)	**0.1**	0.0	0.0	0.0	0.0	2.0	7	17.9
	
	Squash/cordial (l/day)	**0.3**	0.0	0.0	0.0	0.0	1.2	21	53.8
	
	Total tap water intake (l/day)	**1.5**	0.0	0.7	1.4	2.2	4.2	36	92.3
	
	Bottled water (l/day)	**0.2**	0.0	0.0	0.0	0.0	4.0	7	17.9
	
	Total fluid intake (l/day)	**1.6**	0.2	0.8	1.4	2.4	4.2	39	100.0


**WORK**	Tap water (filtered and unfiltered) (l/day) *	**0.2**	0.0	0.0	0.0	0.4	2.0	6	28.6
	
	Filtered tap water (l/day) *	**0.0**	0.0	0.0	0.0	0.0	0.0	0	0.0
	
	Unfiltered tap water (l/day) *	**0.2**	0.0	0.0	0.0	0.4	2.0	6	28.6
	
	Tea (l/day) *	**0.2**	0.0	0.0	0.0	0.2	1.0	6	28.6
	
	Coffee (l/day) *	**0.1**	0.0	0.0	0.0	0.0	0.8	5	23.8
	
	Squash/cordial (l/day) *	**0.1**	0.0	0.0	0.0	0.0	0.8	2	9.5
	
	Total tap water intake (l/day) *	**0.6**	0.0	0.0	0.4	0.8	2.8	15	71.4
	
	Bottled water (l/day) *	**0.3**	0.0	0.0	0.0	0.6	2.0	8	38.1
	
	Total fluid intake (l/day) *	**0.9**	0.0	0.4	0.8	1.0	2.8	18	85.7


**ELSEWHERE**	Tap water (l/day)	**0.0**	0.0	0.0	0.0	0.0	0.0	0	0.0
	
	Tea (l/day)	**0.0**	0.0	0.0	0.0	0.0	0.0	0	0.0
	
	Coffee (l/day)	**0.0**	0.0	0.0	0.0	0.0	0.0	0	0.0
	
	Squash/cordial (l/day)	**0.0**	0.0	0.0	0.0	0.0	0.0	0	0.0
	
	Total tap water intake (l/day)	**0.0**	0.0	0.0	0.0	0.0	0.0	0	0.0
	
	Bottled water (l/day)	**0.0**	0.0	0.0	0.0	0.0	0.2	1	2.6
	
	Total fluid intake (l/day)	**0.0**	0.0	0.0	0.0	0.0	0.2	1	2.6


**ALL**	Total tap water intake (l/day)	**1.8**	0.0	1.0	1.4	2.4	5.8	37	94.9
	
	Total fluid intake (l/day)	**2.1**	0.4	1.2	1.8	2.7	5.8	39	100.0

	**2b: Showering, Bathing, Swimming**	**Mean**	**Min**	**Percentile Distribution**			**Max**	**Activity carried out**	
						
	**Variable**			**0.25**	**0.50**	**0.75**		**n**	**%**

**SHOWERING & BATHING**	No. showers per week	**5**	0	3	5	7	14	34	87.2
	
	Duration per shower (min) †	**16**	5	10	15	20	60		
	
	Showering (min/week)	**74**	0	35	60	100	300	34	87.2
	
	No. baths per week	**2**	0	0	2	3	7	26	66.7
	
	Duration per bath (min) ‡	**40**	10	26	30	38	120		
	
	Bathing (min/week)	**72**	0	0	60	120	360	26	66.7
	
	Total time showering/bathing (min/week)	**146**	35	73	110	185	540	39	100.0


**SWIM**	No. swimming sessions per week	**1**	0	0	0	0	2	6	15.4
	
	Duration per swim (min) §	**53**	10	26	53	60	120		
	
	Swimming (min/week)	**10**	0	0	0	0	120	6	15.4

* amongst those who were employed (n = 21), † amongst those who reported at least one shower per week (n = 34), ‡ amongst those who reported at least one bath per week (n = 26), §amongst those who reported going swimming at least once per week (n = 6)

#### Home

Total tap water intake at home averaged 1.5 l/day. The largest component of total tap water intake at home came from cold tap water (50.7%), followed by tea (23.1%) and then squash (18.9%). The majority of cold tap water intake was unfiltered (73.1%). 7.7% of women reported no tap water intake from any source at home.

#### Work

Amongst employed women, total tap water intake at work averaged 0.6 l/day. All tap water consumed at work was unfiltered. The largest component of total tap water intake at work came from cold tap water (43.1%), followed by tea (29.3%) and then coffee (17.2%). Women consumed similar quantities of cold tap water and bottled water at work. 28.6% of employed women reported no tap water intake from any source at work.

### Showering & bathing

Showering was reported by 87.2%, and bathing by 66.7%, of women (Table 2(b) ). Amongst those women who reported showering mean duration per shower was 16 minutes. Mean duration of bath was 40 minutes, amongst those reporting bathing. Bath duration tended to be longer than shower duration, but overall time spent showering or bathing per week was similar for both activities.

### Swimming

Only 6 women (15.4%) actually reported going swimming at least once a week. Amongst these women, average duration of swimming session was 53 minutes.

### Water use stratified by demographic characteristics

#### Age

No clear monotonic trends were observed for water consumption across age groups, although there were differences between groups for intakes of total tap water at home (p = 0.01), total tap water overall (p = 0.02) and bottled water (p = 0.05) (see Additional file 1). Duration per shower and total time spent showering and bathing per week were inversely correlated with age (Spearman's correlation: -0.39 (p = 0.02) and -0.36 (p = 0.03) respectively).

#### Employment status

There were no differences in tap water consumption overall, or at home, according to employment status. Duration per shower was significantly longer for unemployed than for employed women (p = 0.04).

#### Income

No clear monotonic trends were observed across income categories, although differences were observed for total tap water intake at work (p = 0.001) and duration per shower (p = 0.02).

#### Ethnicity

When stratifying by ethnicity, the results suggest women of South Asian origin may consume more tap water than women in other ethnic groups, and may spend longer showering and bathing, however differences between groups only reached statistical significance for tap water intake at home (p = 0.02), total tap water intake at work (p = 0.02), and duration per shower (p = 0.02).

Differences between the subgroups may exist for other water use variables, but they did not reach statistical significance.

## Discussion

These results show that cold tap water and tap water based beverages constitute a major part of daily fluid intake for pregnant women, and that the majority of tap water intake occurs at home for both unemployed and employed women. However, for employed women some tap water ingestion occurs at work and this should be considered in DBP exposure assessment. Many previous studies on DBPs and adverse birth outcomes have assessed exposure only at the mother's home, e.g. by using trihalomethane concentrations in the water supply of the mother's residence at time of birth. If, as our study suggests, the majority of tap water intake occurs at home, potential exposure misclassification from excluding exposures at other locations should be relatively small.

Water exposures in our study were higher than reported by the only other UK study on water use by pregnant women. Kaur *et al. *[8] found overall total tap water intake to be 1.31 l/day (calculated from their reported consumption per week), and that women spent 54.3 min/week showering and 54.7 min/week bathing. Barbone *et al. *[9] report total tap water intake of 0.6 l/day in Italy, whilst in the US, Shimokura *et al. *[10] report 0.78 l/day and Zender *et al. *[11] 3.4 l/day. Forssén *et al. *[12] report 120 min/week showering amongst pregnant women in the US, which is greater than we found, but bathing was less at 50 min/week.

Our results suggest that there may be some differences for tap water intake and showering/bathing behaviour according to age, employment status, income and ethnicity. Tap water intake has previously been shown to differ by ethnicity [12] and showering and bathing by ethnicity [13] and socioeconomic status [12]. However, as we found no clear-cut patterns, these factors need further investigation in a larger group of women from the birth cohort. It is important to understand these differences in water behaviour, because maternal age, socioeconomic status, and ethnicity are associated with fetal growth and low birth weight [14-16], and may act as confounders if they are also independently associated with exposure to water. In studies using individual-level data these factors tend to be adjusted for. However, many studies on DBPs and adverse birth outcomes have relied upon exposure assessment and confounding data at an ecological level [17,18] or, due to their retrospective design, information on confounders of interest has been incomplete [19,20]. Consequently, a number of epidemiological studies to date in this field of research have been unable to fully adjust for potential confounding. Interpretation of results from these studies is, therefore, limited by the possibility of residual confounding. The prospective cohort design and comprehensive data collection of Born in Bradford will address these methodological weaknesses and in time help to inform the evidence base about the potential effects of DBPs on birth outcomes.

This study has a number of limitations. The results in this study are based on small numbers of women in one city and may not therefore be generalisable to the wider population of pregnant women. Nonetheless, given that there is very little information available on water use by pregnant women in the UK, we believe that these results are useful as approximate estimates of water use in pregnancy and indicate issues that should be considered in epidemiological studies of DBPs, e.g. potential differences in water use in relation to ethnicity.

There is potential for selection bias in this subset. Due to the prohibitive cost of translation, recruitment to the subset excluded the 12-15% of women who spoke no English. This may explain the greater proportion of women of White British origin and lower proportion of women of Pakistani origin compared to the main cohort. With regards to age, marital status, parity and smoking the nested subset was similar to the main cohort. However, the nested subset appeared better educated and had a greater proportion of women in higher income brackets than the main cohort. Thus it is possible that our results may not fully reflect water use in women with lower levels of educational attainment or income.

## Conclusion

This study provides estimates of water exposure in pregnant women in a multi-ethnic population in the north of England. The findings are valuable to inform exposure assessment in studies assessing the relationship between DBPs and adverse birth outcomes. Future work will involve further investigation of potential differences between demographic subgroups on a larger dataset, using regression-type analyses, and validation of questionnaire responses for water exposures. This will be undertaken by comparing questionnaire responses with records of water use kept by the 39 women in a 7-day exposure diary.

## List of abbreviations used

DBPs: Disinfection By-Products

## Note

The peer review of this article can be found in Additional file 2.

## Competing interests

The authors declare that they have no competing interests.

## Authors' contributions

JW is the chief investigator for the Born in Bradford study and participated in the design and implementation of this study. PR participated in the design and implementation of the Born in Bradford study and provided oversight and supervision of recruitment processes for Born in Bradford and the nested study. MJN and MBT originated and designed the nested study. RBS participated in design and implementation of the nested study. RBS and MBT recruited women to the nested study. RBS performed the statistical analysis and drafted the manuscript. MBT and MJN provided critical input to analysis and manuscript. All authors read and approved the manuscript.

## Supplementary Material

Additional file 1 **Water Use stratified by Demographic Characteristics**. Results Table. A table showing water use variables stratified by age, employment, income and ethnicity categories, and significance tests for differences between the various subgroups. Click here for file

Additional file 2 Peer review. Click here for file
