# Supplementary material for: Tap water use amongst pregnant women in a multi-ethnic cohort
Source: Environ Health. 2009 Dec 21;8(Suppl 1):S7. doi: 10.1186/1476-069X-8-S1-S7 (PMC2796503; doi:10.1186/1476-069X-8-S1-S7)
Supplement: Additional file 2 — Peer review. [file 1476-069X-8-S1-S7-S2.doc]

| Article ref no. | **A1471-2105X SZ S7** |
| --- | --- |
| Title | **Tap Water use amongst pregnant women in a multi-ethnic cohort** |
| Author(s) | Smith, Rachel B et al |

**Ref#1**

**General comments:**

Exposure assessment is based upon a questionnaire. It is therefore not clear as to how accurate the data is regarding actual consumption of tap water, bathing and showering. Is it therefore acceptable to report this to one decimal place?

***The authors accept this comment and have adjusted the results in the paper accordingly. Consumption of tap water is now reported to one decimal place, not two (i.e. to the nearest 100 millilitres, rather than the nearest 10 millilitres). Durations of showering and bathing are now reported the nearest whole number (i.e. to the nearest minute). Number of showering, bathing and swimming events per week are reported to the nearest whole number.***

The numbers recruited are small so it is therefore difficult to interpret with confidence.

***The authors acknowledge that the numbers in the analysis are small and that the results should be interpreted with a degree of caution. Nonetheless, given that there is very little information available on water use by pregnant women in the UK (just one study by Kaur et al), we believe that this information is a useful contribution to the literature, in spite of its limitations. We acknowledge that our sample size means it is difficult to be precise, but our results indicate areas of interest for exposure assessment in epidemiological studies of DBPs/THMs and reproductive outcomes, e.g. differences in water use in relation to ethnicity. We have added a comment to this effect in the Discussion section on page 9 in the revised version of this paper. Four of the studies with which we compare our results with in the discussion (Kaur et al, Barbone et al, Zender et al and Forssen et al) are based on larger sample sizes than ours, although that by Shimokura et al reports water use in pregnant women based on a sample size of 34.***

It is assumed that this cohort is exposed to THMs, although it is noted that concentrations in tap water have not been measured per se.

***The water company in the study area has kindly provided the authors with routinely collected information on THM concentrations in tap water, and as part of the HiWATE project we have also conducted extra tap water sampling in the study area for non-THM disinfection by-products. We therefore have information on disinfection by-product (DBP) concentrations in tap water which will be used to estimate DBP exposure for each mother in the main cohort. This is the next phase of our work. Due to word limit constraints we had not mentioned this in the original version of this paper; however we have now added a comment in the Background section on page 3 in the revised paper to make it clear we have this data.***

There are many potential confounders, including smoking and alcohol consumption, whilst there is evidence (albeit equivocal) to suggest that living in the proximity of a landfill may be implicated in con mals and low birth weight. All these factors need to be considered.

***The authors agree that there are many potential confounders of the relation between DBP exposure and birth weight. We will have access to a wide range of individual level data on potential risk factors for low birth weight, e.g. smoking, alcohol and drug consumption during pregnancy, indicators of socio-economic status, educational achievement, ethnicity, occupation, and medical history. This information has been collected prospectively by the Born in Bradford baseline questionnaire and extracted from medical records systems and will be available for inclusion in analyses of the association between birth outcomes and exposures.***

***With particular reference to the reviewer’s comment re proximity to landfill. This particular issue does not form part of our environmental exposure remit for this cohort. Whilst landfill proximity may be associated with low birth weight, we think it unlikely to also be associated with exposure to DBPs and therefore to be a true confounder of the particular relation we are investigating. Given the difficulties inherent in environmental exposure assessment, no study can reasonably be expected to assess exposure to all potential environmental hazards or factors, although potential confounders which cannot be adjusted for should be acknowledged.***

I, therefore, have significant reservations about the validity of this paper.

**Ref#2**

**General comments:**

This is a nice concise descriptive paper, which I enjoyed reading. It’s well written, clear in its methods, thorough and a useful contribution to the literature. There is really very little wrong with it! I have suggested three changes which I think will just tighten the paper up a little and satisfy the readership that all possible angles have been covered.

**Major compulsory revisions:**

None

**Minor essential revisions:**

I think the issue of / potential for sample bias is not sufficiently acknowledged. The sample selected for the study was English speaking, seemed very well educated and had low levels of smoking (though this is of course, a cohort of pregnant women! It would be good to see any differences between the sample and the wider cohort acknowledged and reflected upon. If the sample is representative of the cohort, fine. However, if it’s not representative, the implications of this need to be considered.

***The authors acknowledge that there is potential for selection bias in this sample, but did not address this in the original paper due to constraint on the word limit.***

***Bearing in mind that recruitment to the Born in Bradford main cohort is still ongoing, and at the time of writing the paper access to baseline questionnaire data for the main cohort was not available, we were unable to formally assess how representative the subset was. We have now been able to look at an extract of data from the main cohort (4070 women recruited so far) as per the revised Table 1. With regards to age, marital status, parity and smoking the nested subset was fairly similar to the main cohort. The nested subset was better educated and had a higher proportion of women in higher income brackets than the main cohort. The nested subset contained a higher proportion of women of White British origin and lower proportion of women of Pakistani origin compared to the main cohort.***

***Mirpuri is the main language spoken by women of Pakistani origin in Born in Bradford, and has no written form. Due to the prohibitive cost of translation we could only recruit English speaking women to the subset. This may explain the proportions of women of White British origin and Pakistani origin observed in the subset compared to the main cohort.***

***We have added a column to Table 1 for comparison with the main cohort (recruited so far) and a paragraph to the Discussion section acknowledging the possibility of selection bias and its implications on page 9 in the revised version of this paper.***

**Discretionary revisions:**

First, the sample is described as having been part of a validation sample, but it’s not really clear how this work validates the cohort (or anything really…) Can the authors clarify this part of the work?

***Our overall aim is to assess exposure to DBPs during pregnancy, and examine the association between DBP exposure and outcomes such as low birth weight, within the Born in Bradford cohort. The subset of women were recruited as part of a validation study nested with the main cohort. All women in the subset completed a water use and time-activity diary for 7 days, provided a urine sample for biomarker analysis, and carried out personal monitoring of NO_2_. This information has been collected to validate exposure assessment of water use/DBP exposure and air pollution within the Born in Bradford cohort.***

***We had access to baseline questionnaire data for the women in this validation subset, in order to carry out validation comparisons with the diary and urine biomarker. We are currently in the process of finalising our validation analyses on this subset.***

***However, the intention of this paper was to report the initial descriptive analysis of water use according to the baseline questionnaire within this subset. Given that there is currently very little information on water use by pregnant women in the UK, we believe this to be a useful contribution the literature.***

***We have modified the Methods section on page 4 in the revised version of this paper, to clarify this matter.***

Second, I imagine that the sample chosen was too small to conduct regression-type analyses, but it would useful if the authors either acknowledge this, or if the sample would have permitted models and interaction analyses etc., the authors could usefully explain why they haven’t pursued this approach.

***We did not attempt regression-type analyses simply because the sample size was too small. This descriptive analysis was not our main objective when recruiting the subset, but we found our initial descriptive analyses of the questionnaire to be informative. The findings from the stratified analysis have suggested areas that may be particularly important to examine using regression analyses when we have access to a larger sample of data. Of particular interest are water use and ethnicity, and the implications any relation between these factors may have upon the interpretation of results from epidemiological studies which have been unable to adjust for ethnicity.***

***We have added a comment, indicating our future intention to carry out regression analyses on a larger dataset, to the Conclusion section on page 10 in the revised version of this paper.***
